# A Blueprint for a Mutationist Theory of Replicative Strand Asymmetries Formation

**DOI:** 10.2174/138920212799034730

**Published:** 2012-03

**Authors:** Vladislav V Khrustalev, Eugene V Barkovsky

**Affiliations:** Department of General Chemistry, Belarussian State Medical University, Belarus, Minsk, Dzerzinskogo, 83, Russia

**Keywords:** Chirochore, GC-content, isochore, mutational pressure, nonsense mutation, replichore.

## Abstract

In the present review, we summarized current knowledge on replicative strand asymmetries in prokaryotic genomes. A cornerstone for the creation of a theory of their formation has been overviewed. According to our recent works, the probability of nonsense mutation caused by replication-associated mutational pressure is higher for genes from lagging strands than for genes from leading strands of both bacterial and archaeal genomes. Lower density of open reading frames in lagging strands can be explained by faster rates of nonsense mutations in genes situated on them. According to the asymmetries in nucleotide usage in fourfold and twofold degenerate sites, the direction of replication-associated mutational pressure for genes from lagging strands is usually the same as the direction of transcription-associated mutational pressure. It means that lagging strands should accumulate more 8-oxo-G, uracil and 5-formyl-uracil, respectively. In our opinion, consequences of cytosine deamination (C to T transitions) do not lead to the decrease of cytosine usage in genes from lagging strands because of the consequences of thymine oxidation (T to C transitions), while guanine oxidation (causing G to T transversions) makes the main contribution into the decrease of guanine usage in fourfold degenerate sites of genes from lagging strands. Nucleotide usage asymmetries and bias in density of coding regions can be found in archaeal genomes, although, the percent of “inversed” asymmetries is much higher for them than for bacterial genomes. “Homogenized” and “inversed” replicative strand asymmetries in archaeal genomes can be used as retrospective indexes for detection of *OriC* translocations and large inversions.

## 1 INTRODUCTION

It is well known that the number of open reading frames is higher in leading strands of bacterial “chromosomes” than in lagging strands. This fact has been described in many articles [1, 2] and books [3]. Strong bias to coorient transcription and replication was thought to be due to selective pressure for processive, efficient, and accurate replication [4]. Preferential positioning of bacterial genes in the leading strand should make the high head-on collision rates between DNA and RNA polymerases lower [5]. Rocha and Danchin [6], however, showed that in *Bacillus subtilis* and *Escherichia coli* essentiality of the transcript product, and not expressiveness, selectively drives the biased gene distribution.

In our recent work [7], we found out that the probability of nonsense mutation occurrence is higher for genes situated on lagging strands of DNA. It means that mutations occurring during replication are causing nonsense mutations in genes from lagging strands more frequently than in genes from leading strands. This finding is in consistence with the observation of Mackiewicz *et al*. [8] that the number of gene copies is always higher on leading strands of bacteria than on lagging ones.

Another replicative strand bias is in the nucleotide content distribution between leading and lagging strands. It is generally believed that leading strands of bacterial genomes are enriched with guanine, while lagging strands are enriched with cytosine [9, 10]. This bias is partially connected with the first one, since most of the coding regions contain more codons with guanine situated in first positions than those with cytosine [11, 12]. It means that the higher is the density of coding regions in leading strand, the higher is the total usage of guanine inside it [3]. That rule of nucleotide content distribution in codon positions seems to be conserved in bacteria, archaea and even in eukaryotic viruses [11, 13]. It seems to be a kind of feature inherited by all the protein coding genes derived from their ancient common predecessor. This feature makes empiric Szybalski's rule obey. Szybalski's rule states that the usage of adenine and guanine (purine nucleotides) is always higher in leading strand than in lagging strand [14]. Szybalski's rule may not obey only in highly GC-rich genomes, in which mutational transcription-associated C-pressure and asymmetric negative selection on amino acid substitutions occurring due to GC-pressure may lead to the strong cytosine-loading of coding regions [11].

For the most of bacterial genomes guanine content in fourfold degenerate sites (G4f) is higher in genes from leading strands than in genes from lagging strands, while cytosine content in those sites (C4f) is lower [15]. In the same time, thymine content is higher in fourfold degenerate sites (T4f) from genes situated in leading strands [15], while adenine content (A4f) is lower. Interestingly, similar asymmetries are characteristic to bacterial plasmids that have functional *OriC* region [16, 17]. Many reasons for that bias have been suggested by different researchers [18, 19]. It was suggested that there has been an evolutionary selection pressure for the purine-loading of RNAs [3, 14, 18].

However, in our opinion, one should not look for the reason of biological event if one can easily suggest the cause of it. Generally speaking, there are usually many consequences of each genetic event which can be suggested to be negative or positive. It is an overestimation to think that positive selection favored formation of the bias in density of coding regions or the bias in nucleotide content “with the aim” to reach only a single consequence from the full set (namely, the decrease in the probability of stem-loop formation by mRNAs with biased purine/pyrimidine ratio [18]).

There are several bacterial genomes with “inversed” asymmetries in both density of coding regions and nucleotide content [7]. The strength of both asymmetries is relatively weak for many of bacterial genomes [1, 7]. Some of the genomes have no clear replicative strand asymmetries at all [7, 20]. All those facts have made us sure that asymmetries in nucleotide content and in coding regions density are consequences of the same cause. That cause is known as asymmetric replication-associated mutational pressure [9, 10, 15, 19]. That term highlights that certain types of nucleotide mutations occur in lagging strands of DNA more frequently than in leading strands [9, 10, 15, 19]. The most of researchers suggest that asymmetries in nucleotide usage associated with replication are formed during the replication itself [1, 10, 15]. However, Chen and Chen [2] described a hypothesis that those asymmetries may be formed by asymmetric mutational pressure occurring during transcription. The higher is the density of open reading frames in leading strand, the longer is the period if its existence in the state of nontranscribed strand during transcription [2]. This mechanism surely works in genomes with strong bias in density of coding regions, while its contribution into asymmetry in nucleotide usage should be low in genomes with weak bias in density of coding regions.

The present review briefly describes our hypothesis of replicative strand asymmetries formation which works well in both bacterial and archaeal genomes.

## 2 RELATIONSHIPS BETWEEN TOTAL GC-CONTENT AND REPLICATIVE STRAND ASYMMETRIES IN BACTERIAL GENOMES

**Fig. (1):**
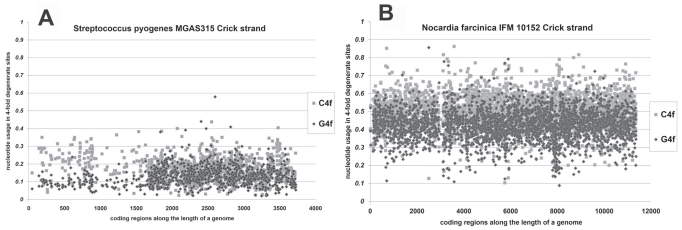
Nucleotide usage in every coding region along the length of (**A**) *Streptococcus pyogenes* MGAS315 Crick strand and (**B**) *Nocardia farcinica* IFM 10152 Crick strand.

Nucleotide usage asymmetries have been revisited by us in 27 bacterial chromosomes of different total GC-content. We confirmed the known fact [15] that the level of G4f is significantly higher (P < 0.001) in genes from leading strands than in genes from lagging strands; average difference is equal to 2.91±0.36%. Level of C4f is significantly lower (P < 0.001) in genes from leading strands than in genes from lagging strands; average difference is equal to 3.54±0.37% [7]. In most of the genomes that bias is strong (see Fig. **1A**), while it is hard to recognize it *ad oculus* in some of them (see Fig. **1B**). In this and other figures “Watson strand” refers to the stand used as a reference in a database (i.e. the "plus" stand) and “Crick strand” refers to its complement [21].

The level of T4f is significantly higher (P < 0.05) in genes from leading strands than in genes from lagging strands; however, an average difference is rather low; it is equal to 1.00±0.50%. There was no significant difference between the level of A4f in genes from leading and lagging strands [7].

Nucleotide usage in twofold degenerate sites from third codon positions (G2f3p, C2f3p, A2f3p and T2f3p) is biased in the similar way as nucleotide usage in fourfold degenerate sites. G2f3p and T2f3p are significantly higher in genes from leading strands, while C2f3p is higher in genes from lagging strands. Interestingly, the difference for A2f3p usage between genes from leading and lagging strands was significant, unlike that for A4f (A2f3p was higher in genes from lagging strands) [11].

In 6 from 12 bacterial chromosomes with G+C lower than 40%, we observed that A4f usage is higher in leading strands, while T4f usage is higher in lagging strands [11]. This observation is in consistence with results obtained by Qu *et al*. [12].

The most probable cause of the decrease in guanine usage in lagging strands is the process of guanine oxidation producing G to T transversions [7, 11]. Oxidation of guanine occurs more frequently in single-stranded DNA than in double-stranded [22]. Indeed, it was shown that oxidation of guanine takes place in nontranscribed strands of DNA (in coding regions) more frequently than in transcribed ones [23, 24]. This circumstance led to the following bias: the level of G4f is often lower than the level of C4f in bacterial and archaeal genes [11]. This kind of bias is stronger for genes from lagging strands of DNA than for those from leading strands [11].

The rates of cytosine deamination are also higher for nontranscribed strands of DNA because they exist in single-stranded state longer than transcribed strands [23, 24, 25, 26]. However, coding regions, in general, contain more cytosine than guanine in their twofold degenerate sites from third codon positions [11]. This bias is not caused by characteristic features of amino acid frequencies distribution [11]. In our opinion, another mutational process (thymine oxidation leading to T to C transitions [28]) should “hide” consequences of C to T transitions in coding regions. Form this point of view, one should expect that cytosine deamination should also occur more frequently in lagging strands of DNA, although cytosine usage is increased and not decreased in lagging strands. However, some authors believe that cytosine deamination happens more frequently in leading strands [27]. Increase in cytosine usage in lagging strands can be explained by i) frequent G to T and not C to A transversions (see above) and ii) frequent T to C transitions [7, 11].

The bias in density of open reading frames was low (more than 40% of genes were situated in lagging strands of each from two replichores) for 7 from 10 genomes with G+C higher than 50% [11]. The example of the weak bias in coding regions density can be seen in Fig. (**1B**). Among 17 bacterial chromosomes with G+C lower than 50% there were only 5 with low difference in density of open reading frames [11].

In 6 from 17 bacterial chromosomes with G+C lower than 50% bias in the density of open reading frames was extremely strong (less than 30% of genes were situated in lagging strands of each from two replichores just like in the case represented in Fig. **1A**), while there was only 1 from 10 genomes with such a strong bias among GC-rich bacteria [11].

Average percent of coding regions in leading strands for 20 GC-rich replichores (with G+C higher than 50%) is equal to 58.6±3.0%. It is significantly lower (p<0.004) than the average percentage of coding regions in leading strands for 34 GC-poor replichores, which is equal to 66.0±3.7%.

Rocha [29] found out that extremely strong bias in density of coding regions is correlated with the presence of class II family C DNA polymerase (PolC) in bacterial genomes. That type of DNA polymerase can be found in genomes of low G+C Gram-positive bacteria (such as *Clostridium*, *Bacillus*, *Lactobacillus*, *Staphylococcus* and *Streptococcus*), as well as in mycoplasmas and thermotogales [30]. Genomes of most of the species possessing PolC are GC-poor [11]. In our opinion, the direct cause of the strong bias in coding regions density is in the ongoing mutational AT-pressure (see section 4) and not in the possession of PolC. It is likely that abovementioned bacterial species inherited PolC enzyme from their common predecessor together with certain characteristic features of their repair system causing mutational AT-pressure. However, those features of repair system have somehow been neutralized in some of the species. For example, it has been shown that *Thermotoga maritima *with genomic G+C equal to 46.1% possesses functional PolC homologue [30]. In one of its replichores just 58% of open reading frames are situated on leading strand, while that percentage for leading strand from another replichore is equal to 50% (see above) [7, 11].

On the other hand, there is a strong bias in coding regions density in the GC-poor genome (G+C=28.4%) of *Borrelia burgdorferi* B31 which does not possess PolC (34 and 33% of open reading frames can be found in lagging strands of its two replichores) [7, 11].

## 3 DEVIATIONS FROM GENERAL RULES OF REPLICATIVE STRAND ASYMMETRIES ARE RARE AMONG BACTERIAL GENOMES AND COMMON AMONG ARCHAEAL GENOMES

In this section, we briefly overviewed several interesting findings that provided us the material for creation of our own hypothesis.

It is known that inversions inside a single replichore locally disturb the pattern of nucleotide usage distribution between leading and lagging strands [31]. It happens because a part of leading strand becomes a part of lagging strand and *vice versa* [31]. For example, several relatively short inversions in one of the replichores of *Thermotoga maritima* MSB8 led to the loss of significant bias in G4f usage between genes from leading and lagging strands. Moreover, those inversions have made the density of coding regions in leading strand of that replichore equal to the density of coding regions in lagging strand [7]. The fact that such deviations are relatively rare among bacterial genomes studied, while inversions should happen frequently in bacterial genomes [31] made us suggest that there is a natural process not only creating but also maintaining replicative strand asymmetries.

In one of the replichores of *Thermus thermophilus* HB27 the bias in nucleotide usage is “correct”, while the density of coding regions in leading strand is just 47% [7]. The cause of this deviation is also in the inversion. Nucleotide usage asymmetries in that inverted region have already been improved, while the density of open reading frames has not.

There are two genomes with “inversed” nucleotide usage asymmetries among bacterial genomes studied by us [7]. Those asymmetries seem to be formed due to translocation of *OriC* region to the area near the *Ter* region. This mutation is rare for bacterial genomes in which site-specific termination of replication exists [32]. Since there is no site-specific termination of replication in archaeal genomes [32, 33], analogous cases are widespread among them [11].

Significant bias in G4f distribution between leading and lagging strands have been found by us in 16 from 25 archaeal genomes with relatively homogenous GC-content distribution along the “chromosome”. In 8 from those 16 genomes replicative strand asymmetries were “inversed”: the level of G4f was higher for the strand with lower density of coding regions.

As to the bias in C4f distribution, its “correct” variant has been found in 11 archaeal genomes; “inversed” variant has been found in 8 genomes. “Correct” asymmetries in A4f and T4f have been found in 13 and 14 genomes, respectively. Only 4 archaeal genomes possess replichores with “inversed” asymmetries in A4f and T4f (in those genomes the usage of T4f is elevated in genes from strands with lower density of open reading frames) [11].

There are just 2 bacterial species (*Aquifex aeolicus* and *Nostoc sp. PCC 7120*) without any sign of replicative strand asymmetries (among 30 that have been studied by us [7]) and 3 archaeal species (*Archaeoglobus fulgidus DSM 4304*, *Nanoarchaeum equitans Kin4-M* and *Picrophilus torridus DSM 9790*) among those 25 genomes with relatively homogenous GC-content distribution along “chromosome” [11].

## 4 PROBABILITY OF NONSENSE MUTATION OCCURRENCE DUE TO REPLICATION-ASSOCIATED MUTATIONAL PRESSURE IS HIGHER FOR GENES FROM LAGGING STRANDS IN BOTH BACTERIAL AND ARCHAEAL GENOMES

**Fig. (2):**
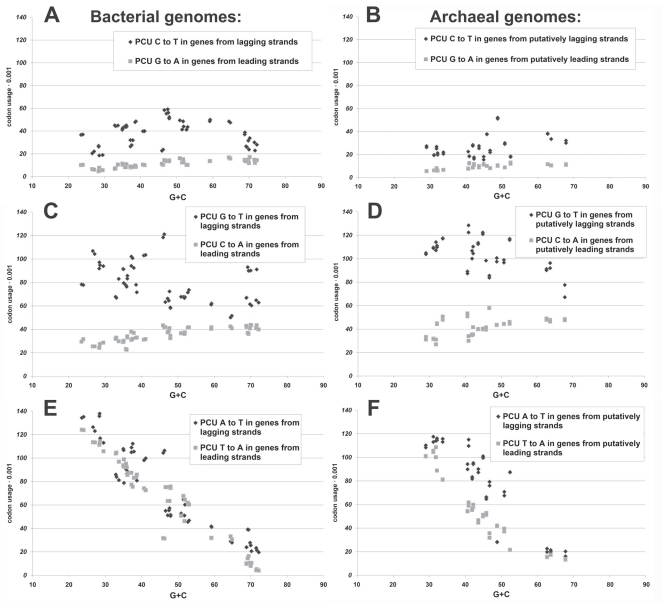
Usage of preterminal codons (PCU) able to become terminal due to certain types of single nucleotide mutations in bacterial (**A**, **C**, **E**) and archaeal (**B**, **D**, **F**) genomes.

Preterminal codon is a codon which may become terminal (stop-codon) due to a single nucleotide mutation [34]. Preterminal codons can be classified into several groups according to the type of nucleotide mutation which may make them terminal [35]. For example, total usage of preterminal codons which may become terminal due to C to T transition is called “PCU C to T”. We have shown [7] that the usage of “PCU C to T” is always higher in coding genomes of bacteria than the usage of “PCU G to A” (see Fig. **2A**). In that article [7], we claimed that this rule should be characteristic to coding genomes of other organisms as well. As one can see in Fig. (**3B**), the usage of “PCU C to T” is really higher than the usage of “PCU G to A” for all the coding genomes of archaea [11], although the difference between them is lower than that in the most of bacterial genomes.

Cytosine deamination resulting in C to T transitions should happen in lagging strands more frequently than in leading strands [7]. C to T mutations occurring in lagging strands are inherited by leading strands as G to A mutations. The usage of the substrate for nonsense C to T transitions is always higher than the usage of the substrate for nonsense G to A transitions. That is why genes situated on lagging strands are at higher risk of nonsense mutation due to C to T transitions frequently occurring in those strands, while genes situated on leading strands are at lower risk of nonsense mutation occurrence due to G to A transitions which are consequences of cytosine deamination in lagging strands [7].

Oxidized guanine (8-oxo-G) may cause G to T transversion. This transversion occurred in lagging strand is inherited by leading strand as C to A transversion. As one can see in Figs. (**2C** and **D**), the usage of preterminal codons which may become terminal due to G to T transversion (PCU G to T) is always much higher than the usage of preterminal codons which may become terminal due to C to A transversion (PCU C to A) in both bacterial [7] and archaeal [11] coding genomes.

The usage of preterminal codons which may become terminal by the way of A to T or T to A transversion is very high in genes from AT-rich genomes, while their usage is low in genes from GC-rich ones. Dependences represented in Figs. (**2E** and **F**) make the main contribution into the inversed correlation between total usage of preterminal codons (PCU) and G+C [35]. As one can see in Figs. (**2E** and **F**), PCU A to T is higher than PCU T to A in most of the archaeal coding genomes, while the number of bacterial genomes with the same difference is lower [7]. Molecular mechanisms for both A to T and T to A transversions have been suggested [36].

Existence of differences in the usages of different groups of preterminal codons can be explained by the common predecessor’s effect and negative selection on amino acid content of proteins. Indeed, low usage of PCU G to A is connected with the low usage of tryptophan in proteomes, since G to A transitions may be nonsense only in a single codon coding for that rare amino acid residue [15, 26, 37]. High usage of PCU G to T usage is connected with relatively high level of glutamic acid usage in proteomes [7].

In general, we can state that cytosine deamination and guanine oxidation frequently occurring in lagging strands during replication should produce more nonsense mutations in genes from lagging strands than in genes from leading strands [7]. This circumstance should lead to the lower density of open reading frames in lagging strands. Since both C to T and G to T mutations being a part of mutational AT-pressure occur more frequently in GC-poor genomes, one should not be surprised that the difference in open reading frames density between leading and lagging strands is often very low in GC-rich genomes [7].

Low GC-content is a kind of retrospective feature making one sure that there was a strong AT-pressure in the predecessor of that specie for a long period of time [38]. However, one cannot be sure that the direction of mutational pressure has not been changed in a recent period of time in the genome of given specie [37]. For example, we have shown that GC-content of the genome of common predecessor of archaea was lower than that for the most of its offspring including those with G+C lower than 50% [37]. According to our hypothesis, strong bias in density of coding regions is the consequence of ongoing mutational AT-pressure. One can observe GC-poor genomes with relatively weak bias in density of coding regions in case if rates of GC to AT mutations have become lower in a recent period of time (which was sufficient for partial reduction of that bias), while total GC-content of that genome is still below 50%. On the other hand, strong bias in density of coding regions can sometimes be found among genomes with GC-content higher than 50% in case if the rates of GC to AT mutations in them have been elevated not very long time ago. Another critical factor for the formation of the strong bias in open reading frames density should be relatively low rates of gene duplication events.

## 5 REPLICATIVE STRAND ASYMMETRIES ARE RETROSPECTIVE INDEXES HELPING TO RECONSTRUCT HISTORY OF *ORIC* TRANSLOCATIONS AND LARGE INVERSIONS IN ARCHAEAL GENOMES

We can state that both asymmetries in open reading frames density and in nucleotide content are being formed in archaeal genomes by replication-associated mutational pressure [11]. They have become clear and “correct” only in relatively stable genomes (in those without large inversions and translocations). Almost perfect replicative strand asymmetries have been found by us in both archaeal genomes with a single *OriC*, and in those with three *OriC* regions [39]. It means that existence of several *OriC* regions is not the cause of the absence of nucleotide usage asymmetries, as it has once been suggested [20]. Translocations, duplications and deletions of *OriC* regions lead to the formation of “inversed” asymmetries. Those “inversed” asymmetries become weaker with each replication cycle. After certain amount of generations “inversed” asymmetries may become unrecognizable. One cannot observe them during the period of time between “inversed” asymmetries disappearance and “correct” asymmetries appearance [11]. Sooner or later altered asymmetries become “improved” by natural mutational process.

Interesting example of nucleotide usage asymmetries “homogenization” have been found by us in the genome of *Methanosarcina acetivorans*. The usage of A4f in genes from Watson strand of DNA is practically equal to that in genes from Crick strand in the area of *Methanosarcina acetivorans* genome designated in Fig. (**3**). There is no significant difference between usages of T4f in genes from Watson and Crick strands in that area of the genome [11]. In certain prokaryotic genomes this kind of “homogenization” is characteristic for the whole “chromosome”. In the genome of *Methanosarcina acetivorans,* one can see two areas with clear asymmetric asymmetries in adenine and thymine usages. In the area from nucleotide 1 to 2 000 A4f is significantly higher in genes from Crick strand than in genes from Watson strand, while T4f is significantly higher in genes from Watson strand. In the area from nucleotide 7 000 to 9 000 genes from Watson strand are enriched with A4f, while genes from Crick strand are enriched with T4f. The simplest explanation of this pattern of nucleotide usage asymmetries is in the duplication of *OriC *region. Newly duplicated *OriC* situated near the region in which two replication forks had met previously should produce “homogenization” of nucleotide usage asymmetries in the half of genome. On the other hand, there might be two *OriC* regions in the genome of *Methanosarcina acetivorans* situated opposite each other. Then one of them might lose its function [11].

**Fig. (4):**
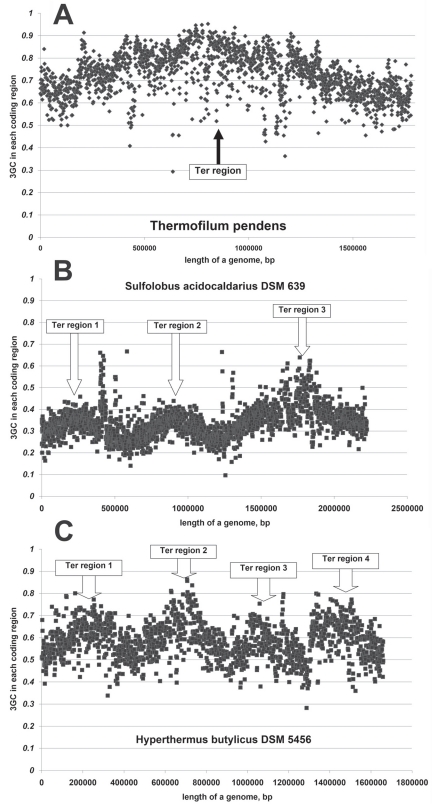
GC-content in third codon positions (3GC) of genes along the length of (**A**) *Thermophilum pendens* Hrk 5; (**B**) *Sulfolobus acidocaldarius* DSM 639 (B) and *Hyperthermus butylicus* DSM 5456 genomes.

Successful prediction of *OriC* location in archaeal genomes usually requires thorough study of both nucleotide usage asymmetries and GC-content distribution along the length of “chromosome” [39]. In our work [39], we found out that in certain archaeal species GC-content in third codon positions of all the 64 codons (3GC) of genes highly depends on the distance between them and *OriC*. The longer is the distance from gene to *OriC*, the higher is the level of 3GC inside it [39]. Interestingly, the situation is quite different for bacterial genomes: the longer is the distance, the lower is 3GC [40]. However, intragenomic difference in 3GC levels caused by replication-associated mutational pressure is usually much higher in archaeal genomes than in bacterial ones [39]. We decided to use a term “protoisochores” [39] to refer to characteristic sinusoid-like pictures of 3GC distribution along the length of certain archaeal genomes (see Fig. **4**). “Protoisochores” have been found in the genome with single *OriC* (*Thermophilum pendens*), in the genome with three *OriC* regions (*Sulfolobus acidocaldarius*), as well as in the genome with four *OriC* regions (*Hyperthermus butylicus*) [41]. Interestingly, clear “replicores” can be found in genomes possessing clear “protoisochores” [39].

**Fig. (5):**
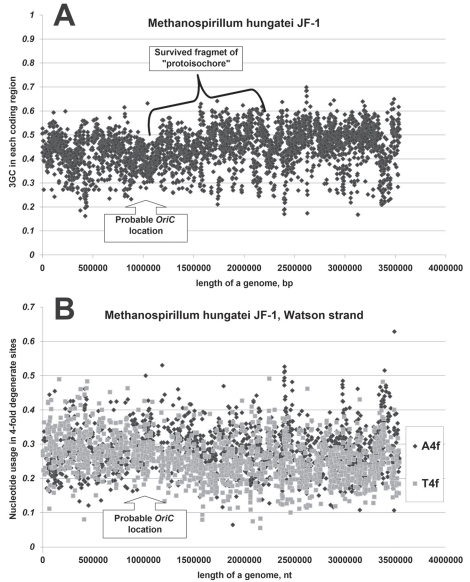
GC-content in third codon positions (3GC) of genes along the length of (**A**) *Methanospirillum hungatei* JF-1 genome and (**B**) nucleotide usage in every coding region along the length of *Methanospirillum hungatei* JF-1 Watson strand of DNA.

*OriC* translocations and long inversions should disturb not only nucleotide usage asymmetries in leading and lagging strands, but also the pattern of 3GC distribution along the “chromosome” [11]. Several archaeal genomes with “remains” of “protoisochores” have been found by us [11, 39]. One of those genomes belongs to *Methanospirillum hungatei* (see Fig. **5**). There is a fragment of “protoisochore” which still can be recognized in Fig. (**5A**). The region around the lowest point of 3GC should contain *OriC* region. As one can see in Fig. (**5B**), asymmetries in A4f and T4f also change their directions in that region. The length of an area with A4f>T4f bias is about 2.5 times longer than the length of an area with T4f>A4f bias for the Watson strand of *Methanospirillum hungatei *genome (see Fig. **5B**) [11]. This pattern of nucleotide usage distribution can be explained by a long inversion. About three quarters of the “left” replichore and only about one quarter of the “right” replichore have been included in that inversion. That is why about a half of the “left” replichore still possesses “inversed” nucleotide usage asymmetries. Looking in Fig. (**5A**) one cannot find a clear peak of 3GC. Instead of it there is a long “plateau” with relatively elevated 3GC usage. Formation of a long “plateau” can also be explained by the hypothesis of a long inversion. The region with 3GC peak appeared closer to the *OriC* after the inversion than it was before. The region of the “left” replichore with low 3GC usage appeared opposite the *OriC*. After the long inversion had happened, 3GC has grown in genes situated in the above mentioned region, while GC-content of genes previously situated in the region with 3GC peak has decreased.

## CONCLUSIONS

In the present review, we focused on a fact which can be a cornerstone for the creation of a theory of replicative strand asymmetries formation. Mutational pressure occurring during replication is able not only to produce nucleotide usage asymmetries between genes from leading and lagging strands, but it is also able to introduce more nonsense mutations into genes from lagging strands than into genes from leading strands [7, 11]. Another important aspect of the review is as follows. Large genomic rearrangements changing position and sometimes quantity of *OriC* regions should be responsible for the formation of “inversed” replicative strand asymmetries, which are much more frequent among archaeal genomes than among bacterial genomes. The absence of replicative strand asymmetries cannot be explained by the existence of multiple *OriC* regions, while it can be explained by their frequent duplications, deletions and translocations [11, 39].

## Figures and Tables

**Fig. (3):**
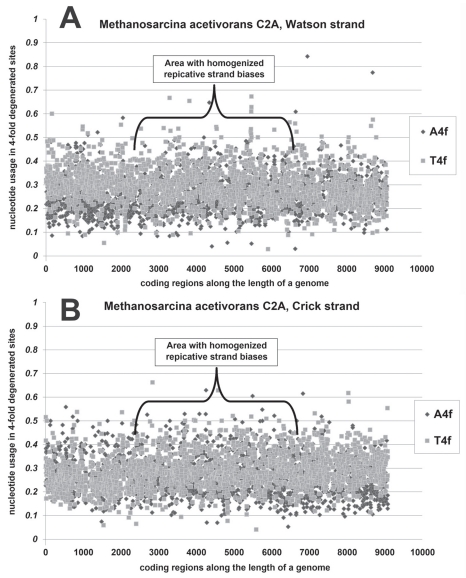
Nucleotide usage in every coding region along the length of *Methanosarcina acetivorans* C2A (**A**) Watson and (**B**) Crick strands of DNA.
